# Primary invasive laryngeal mycosis in an immunocompetent patient: a case report and clinico-epidemiological update

**DOI:** 10.1186/s12879-018-3219-1

**Published:** 2018-07-11

**Authors:** Supram Hosuru Subramanya, Joseph Jillwin, Shivaprakash Mandya Rudramurthy, Krishna Chandra Rijal, Niranjan Nayak, Arunaloke Chakrabarti, Arnab Ghosh

**Affiliations:** 1 Department of Medical Microbiology, Manipal College of Medical Sciences, Pokhara, Nepal; 2 Department of Medical Microbiology, Postgraduate Institute of Medical Education and Research, Chandigarh, India

**Keywords:** Primary laryngeal aspergillosis, *Aspergillus fumigatus*, FFPE-PCR

## Abstract

**Background:**

Laryngeal aspergillosis is uncommon and is usually secondary to pulmonary involvement in immunocompromised patients. Primary laryngeal aspergillosis in immunocompetent individuals is extremely rare, with a few cases documented over the last five decades.

**Case presentation:**

We report a case of primary localised laryngeal aspergillosis in a 21-year-old apparently immunocompetent student. Septate hyphae were observed on histopathology of the laryngeal lesion, which was further confirmed as *Aspergillus fumigatus* after extraction of fungal DNA from formalin fixed paraffin embedded tissue (FFPET) and sequencing. The patient responded well to oral itraconazole therapy over a month.

**Conclusions:**

Since last few decades, cases of primary laryngeal aspergillosis in immunocompetent individuals are on the rise, globally. This is the first case of invasive laryngeal aspergillosis reported in Nepal. The extraction of DNA from tissue and sequencing helps to identify the etiological agent, when culture fails to isolate the fungus.

## Background

Primary fungal laryngitis is commonly attributable to yeasts such as *Candida*, and *Cryptococcus* or fungi are known to cause endemic mycoses like *Blastomyces, Paracoccidioides,* and *Coccidioides*. The mold forms, such as *Aspergillus* and *Mucor*, may involve larynx as secondary pulmonary invasion [1, 2]. Immunocompromisation due to leukaemia, AIDS, severe aplastic anaemia, lymphoreticular neoplasms, or immunosuppressive therapy predispose person to invasive fungal infection [1, 2]. Primary laryngeal aspergillosis in immunocompetent individuals is extremely rare. It often mimics the pre-malignant and malignant conditions (squamous cell carcinoma) of larynx. Fungal laryngitis is usually characterized by sore throat, earache, hoarseness of voice, cough, odynophagia, formation of endolaryngeal and perilaryngeal white plaques, granulation tissue, ulcerations, erythema and edema [3]. Diagnosis and prompt treatment are essential to prevent complications like scarring of the vocal folds, compromised airway due to glottic edema and dissemination of the pathogen. First case of aspergillosis of larynx was reported in 1969 from Pondicherry, South India [4]. Globally, less than 50 cases over the period of last 50 years have been documented. Herein, a case of primary laryngeal aspergillosis in an apparently immunocompetent young adult is reported. To the best of our knowledge, this is the first such case report from Nepal. In this endeavour, we conducted a comprehensive review of literature and analysed all previously reported cases.

## Case presentation

A 21-year young male presented to Manipal Teaching Hospital, Pokhara, with progressive hoarseness of voice for two months and frequent cough with expectoration since one month. He had no history of phonotrauma, apparent immune deficiency, leukaemia, malignant disease, diabetes mellitus, broad-spectrum antibiotics or immunosuppressive therapy, including corticosteroids. He was not habituated to tobacco or alcohol. He did not have any previous history of laryngeal trauma, allergies or mycosis. A general physical examination did not reveal lymphadenopathy or organomegaly. There were no visible lesions or masses in the oral cavity, oropharynx or nasopharyngeal mucosa. His paranasal sinuses and chest X-rays were clear. Routine blood test report was within normal limits. Serological markers for Hepatitis B, C, and HIV were negative and VDRL test was non-reactive.

### Clinical examination and laboratory findings

A direct laryngoscopic examination was performed under general anaesthesia. Videostroboscopy revealed a smooth, diffused whitish spheroid submucosal mass on the anterior surface of the left vocal cord. Vocal cord mobility was normal bilaterally, the airway was adequate, and both subglottis and supraglottis showed normal mucosa.

**Fig. 1:**
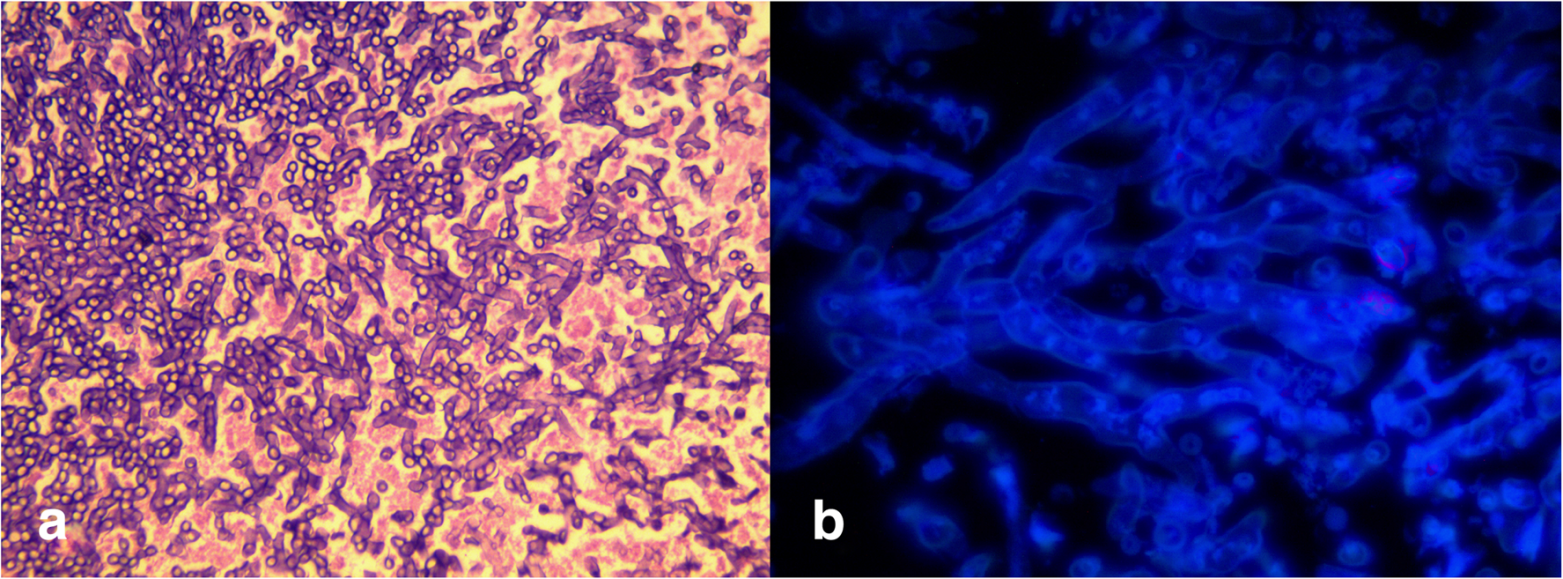
**a** Histopathological features of biopsy sample showing numerous septate hyphae, “spaghetti-like” fungal filaments branching at an angle of approximate 45^0^, interspersed with shreds of vocal cord squamous epithelium (Haematoxylin and Eosin stained, magnification X400. **b** Calcofluor white staining of tissue section observed under fluorescent microscopy showing numerous branched septate hyphal forms X1000

In order to exclude, glottic carcinoma the patient was subjected to punch biopsy from the lesion by the micro-laryngeal procedure under general anaesthesia. The histopathological examination showed conidia and broad septate hyphae, most of them showing acute angle branching without any evidence of malignant cells (Fig. 1). Repeat biopsy specimen processed for fungal culture did not yield any growth. For the identity of fungi, sections from paraffin-embedded tissue block were analysed by Polymerase Chain Reaction (PCR).

### Molecular identification by PCR

#### Extraction of DNA from formalin fixed paraffin embedded (FFPE) tissue

To avoid environmental fungal DNA (or) amplicon contamination, all steps were performed inside laminar air flow cabinets in separate closed cabins equipped with a dedicated set of micropipettes and instruments. A 50 μm thick FFPE tissue section was cut using a clean blade by microtomy and transferred to a 1.5 ml microcentrifuge tube. Deparaffinization and DNA extraction was performed as per Anna Lau et al. [5] with minor modifications where phenol-CHCl_3_-isoamyl alcohol extraction protocol was performed. The pellet was dissolved in 50 μm of nuclease-free water and stored at − 20^0^ C until further testing.

#### Amplification of 28 s region of rDNA

PCR was performed in a 45 μl mixture consisting of 1× PCR buffer without MgCl_2_ (Genei, Bangalore), 2.5 mM MgCl_2_, 0.25 mM deoxyribonucleotide blend (Fermentas), 0.4 μM primers 12F 5’GTTGATAGAAYAATGTAGATAAGG3’ and 13R 5’GACAGTAAGATTCCCCTTG3’ (1) (Eurofins), 1.2 U of Taq Polymerase (Bangalore Genie) and 5 μl (~ 80 ng) of template DNA. Thermal cycling was performed in an Eppendorf Mastercycler Gradient thermal cycler (Eppendorf AG, Hamburg) with the following conditions: denaturation at 95 °C for 10 mins followed by 60 cycles of 94 °C for 15 secs, annealing at 53 °C for 20 secs, and extension 72 °C for 25 secs and finally once at 72 °C for 5 mins. Positive and negative controls were included. Amplification was confirmed by electrophoresis on 2% agarose gel with ethidium bromide, and the amplicon was purified using Qiagen gel extraction kit according to manufacturer’s instructions.

### Sequencing

Bidirectional Sanger sequencing of purified amplicons was performed with the primers mentioned above, using the BigDye Terminator sequencing ready reaction kit (v 3.1) and the products was capillary electrophoresed in an ABI Prism 3130 genetic analyser (Applied Biosystems). Sequences were analysed using Bionumerics software version 7.1 (Applied Maths, Ghent, Belgium and identified through BLASTn (https://blast.ncbi.nlm.nih.gov/Blast.cgi?PAGE_TYPE=BlastSearch). On the basis of comparing the sequences from the specimen with those in the GenBank databases, the fungus in the biopsy was identified as *Aspergillus fumigatus.* Our isolate had 100% matches with the standard strain of ATCC 1022. The sequence data have been deposited in the GenBank database (http://www.ncbi.nlm.nih.gov/Genbank/index.html) with the accession number MH465665.

### Treatment and follow-up of the patient

According to the clinical features and the mycological data, diagnosis of vocal cord aspergillosis was made and the patient was treated with oral itraconazole 200 mg BD for four weeks. Clinical improvement was noticed after a month of antifungal therapy. At the third months’ follow up, his voice had returned to normal, and no residual lesion was seen on laryngoscopy. At the end of 6 months follow up, there was no recurrence.

### Literature review

Extensive search in PubMed/MEDLINE and Google Scholar by two investigators independently, revealed only 38 peer-reviewed reported cases of primary laryngeal aspergillosis in immunocompetent subjects (Table 1).

**Table 1:** Details of 38 cases of primary laryngeal Aspergillosis in immunocompetent patients reported over the period of 50 years, (English literature)

Case	Reference	Age/gender	Geographical area	Clinical presentation	Initial diagnosis	Associated factors	Diagnosis method	Fungal culture	*Aspergillus* species involved	treatment	Follow up period and Outcome
1	Rao PB. 1969	48/M	Pondicherry, S. India	Hoarseness of voice	NA	None	HE	No	NA	No treatment	2 M-asymptomati
2	Ferlito A. et al. 1974	76/M	Verona, Italy	Hoarseness of voice	NA	None	HE	No	NA	No treatment	2 M- asymptomatic
3	Kheir SM et al. 1983	50/M	Birmingham, UK	Hoarseness of voice	Malignancy	COPD	HE and Immunofluorescence studies	No	NA	Topical nystatin powder	24 M- asymptomatic
4	Benson-Mitchell R et al. 1994	62/M	London, UK	Hoarseness of voice	Malignancy	None	HE	No	NA	No treatment	2 M- asymptomatic
5	Nong D, et al. 1997	30–40 4 M + 4F	China	Hoarseness of voice leading to aphonia, sore throat,	Acute laryn giti, TB, malignancy	None	NA	NA	NA	NA	NA
6	Beust L, et al.. 1998	53/M	Rennes, France	Hoarseness of voice, respiratory distress	None	None	HE	No	NA	Laryngectomy	3 M- asymptomatic
7	Fairfax AJ, et al. 1999	75/M	Staffor, UK	Hoarseness of voice, aphonia	None	None	HE and culture	Yes	*A. fumigatus*	AMP-lozenges, 10 mg-4 W	1 M- Improved
8	Dean CM, et al. 2001	17/F	Philadelphia, USA.	Hoarseness of voice, vocal fatigue	None	NG	NA	NA	NA	NA	NA
9	Ogawa Y, et al. 2002	73/M	Tokyo, Japan	Hoarseness of voice (History of Radiotherapy and DB)	Malignancy	None	HE, surgery	NO	NA	Oral ITCZ- 8 W and AMP-B gargle	2 M- No recurrence
10	Wittkopf J, et al. 2006	62/F	Iowa, USA	Fluctuating hoarseness	True vocal fold cyst (? aspergilloma)	Type II DB (well-controlled), hypertension, and GERD	HE	No	NA	Surgery	NA- No recurrence
11	Ran Y, et al. 2008	36/F	China	Hoarseness of voice, vocal fatigue	None	Dexamethasone therapy for rhinitis and asthma	HE, KOH, SEM and Culture	Yes	^a^ *A fumigatus*	Oral ITCZ (200 mg bd-4 W)	1 M- asymptomatic
12	Liu YC, et al. 2010	30/F 32/F	Hangzhou, China	Hoarseness of voice	True vocal cord cyst	Vocal abuse, broad spectrum antibiotic therapy	HE and Culture and FFPE-PCR	Yes (no growth)	^a^ *A. fumigatus*	Oral ITCZ (200 mg bd- 4 W)	1 M- asymptomatic
13	Ran Y, et al. 2011	30/F	Chengdu, China	Hoarseness of voice, vocal fatigue, expectoration, and occasional vomiting	Laryngitis	Vocal abuse, oral Antibiotics and dexamethasone use	HE, KOH, SEM and Culture	Yes	^a^ *A fumigatus*	Oral ITCZ (200 mg bd- first 2 W, 200 mg qd next 2 W	1 M- asymptomatic
14	Sundarray C et al. 2011	NA	Cuttack, India	NA	NA	NA	NA	NA	NA	NA	NA
15	Ran Y, et al. 2013	23/F	China	Hoarseness of voice, severe paroxysmal cough, tachypnea	None	Oral sex	HE, SEM and Culture	Yes	^a^ *A. fumigatus*	Oral ITCZ (200 mg bd- 4 W	1 M- asymptomatic
16	Doloi PK, et al. 2014	35/F	Assam, India	Hoarseness of voice, cough	None	Keratosis of the larynx	HE, KOH and Culture	Yes	*A. fumigatus*	Oral ITCZ (100 mg qd- 3 W)	3 W- asymptomatic
17	Al-Ogaili Z, et al. 2014	77/F	Australia	Dysphagia and hoarseness	Lymphoma	Smoking,inhaled corticosteroids	HE and Culture	Yes	Not speciated	NA	NA
18	Gangopadhyay M, et al. 2014	42/M	West Bengal, India.	Hoarsenes, fever, cough with expectoration	Malignancy	Smoking, vocal abuse	HE, and Culture	Yes	*A. niger*	Oral ITCZ	18 M- asymptomatic
19	Ravikumar et al. 2014	34/F 52/F 38/M	Tamil Nadu, India	Hoarseness, cough, Dysphagia, vocal fatigue	None	NA GERD	HE and KOH mount	No	NA	Oral ITCZ (100 mg bd- 3 W)	3 W- asymptomatic
20	David et al. 2014	59/F	Sydney, Australia	Hoarseness of voice	None	Asthma- fluticasone therapy	HE	No	NA	Oral ITCZ	NA: No recurrence
21	M Dutta, et al. 2015	45/F	WB, India	Hoarseness of voice	Malignancy	None	HE, KOH and Culture	Yes	*A. fumigatus*	Oral ITCZ (300 mg qd-3 W)	6 M- asymptomatic
22	*JCR Villanueva,* et al. *2015*	28/F	Philippines	Hoarseness of voice	Antibiotics and steroids	None	HE	No	No	Oral VCZ (400 mg qd– 4 W)	1 M- asymptomatic
23	Arpita Saha, et al. 2015	28/F	Odisha, India	severe dysphonia	None	Asthma, long-term steroid inhaler, vocal abuse, broadspectrum antibiotics	HE, and Culture	Yes	*A. fumigatus*	VCZ (200 mg bd-8 days)	2 W- asymptomatic
24	Santosh Kumar Swain et al. 2016	35/M	Orissa, India	Hoarseness of voice	Flute player	Malignancy	HE, KOH and Culture	Yes	*A. fumigatus*	Oral ITCZ- 100 mg bd- 3 W	6 M- asymptomatic
25	Richard H. et al.. 2016	73/F	USA	persistent hoarseness	None	Inhaled and oral corticosteroids, and nebulized tobramycin	HE	NO	*NA*	Oral ITCZ-20 W	5 M- asymptomatic
26	Santosh Kumar et al. 2017	12/M	India	Hoarseness of voice	None	Asthma, inhaled corticosteroids, microlaryngeal surgery with stripping of the vocal cords	HE, and Culture	Yes	*A. fumigatus*	Oral ITCZ-50 mg bd- 3 W	3 W- asymptomatic
27	Soumen Chatterjee et.al. 2017	43/F	India West Bengal	Hoarseness of voice	None	None	HE, KOH and Culture	Yes	*A. fumigatus*	Oral ITC -100 mg bd- 8 W	1 M- asymptomatic
28	Present study	22/M	Nepal	hoarseness and frequent expectoration	None	None	HE, KOH, Culture and FFPET-PCR assay	Yes	^a^ *A. fumigatus*	Oral ITCZ- 4 W	1 M- asymptomatic No recurrence

Legend: ^a^Species identified via sequencing; *HE* Histopathological examination, *COPD* Chronic obstructive pulmonary disease, *ITCZ* Itraconazole, *W* Weeks, *M* Month, *AMP-B* Amphotericin B, *DM* Diabetes mellitus, *GERD* Gastroesophageal reflux disease, *SEM* scanning electron microscopy, *VCZ* Voriconazole

## Discussion

### Aetiology of laryngeal aspergillosis

Primary invasion of the larynx by *Aspergillus* is uncommon and is very rare in immunocompetent individuals. As per the literature review, till date, 38 cases of primary laryngeal aspergillosis in immunocompetent patients have been documented over 50 years. *Aspergillus fumigatus* was reported to be the underlying causative mould in the majority of cases, documented so far; except for two cases. *A. niger* infection reported by Gangopadhyay et al. from India [6] and Gallo and colleagues from Italy reported *Aspergillus flavus* as the etiologic agent in a patient with Felty’s syndrome [7]. In the present case a immunocompetent student had *Aspergillus fumigatus* responsible for the laryngeal pathology.

### Disease pathogenesis

*Aspergillus* is a well-known opportunistic fungus causing allergic and invasive disease in immunocompromised hosts [8]. The pathogenesis of laryngeal aspergillosis in an immunocompetent host is not well understood. The *Aspergillus* conidia are ubiquitous in nature as the fungus grows in a saprophytic environment (soil and decaying matter), it could well be possible that exposure of heavy fungal load in air may allow the fungus colonise the dark airway cavities [3] which could favour their slow germination without any symptoms. Such colonisation of the paranasal sinuses leads to fungal ball formation [9]. Hoarseness of voice was the only symptom in this case without any other predisposing conditions like corticosteroid or systemic antibiotic therapy. This is in contrast with other reports where patients developed symptoms after being treated with corticosteroid or systemic antibiotic therapy or after vocal abuse for many years (Table 1).

### Epidemiology and risk factors for developing primary laryngeal aspergillosis

Amongst the reviewed 38 reported cases 16/37 (44%) were males, and 21/37 (57%) were females. Age group ranged from 12 to 74 years. Dutta M et al. [3] reported in 2015 that 50% of immunocompetent subjects with primary laryngeal aspergillosis had no identifiable contributory factors, but 14.29% had vocal abuse and steroid intake. Smoking, broad-spectrum antibiotics and exposure to radiation was detected in 10.7% of cases. Of the cases, 7.1% had vocal fold cyst, whereas 3.6% had a history of COPD, oral sex and diabetes. Six cases have been reported between 2015 and 2017 (Table 1); three of these cases were without any identifiable risk factors. Remaining three cases (and a few documented prior to 2015) had a history of using corticosteroid inhalers for bronchial asthma, which could have led to abrogation of the local immunity in the throat or could have altered the flora of the laryngeal mucosa, allowing the overgrowth of *Aspergillus* [10]. The exact predisposing conditions contributing towards the disease progression in the present case remains obscure but could be multifactorial with a complex interplay between host and the environment.

### Time trend and geographical distribution of primary laryngeal aspergillosis

**Fig. 2:**
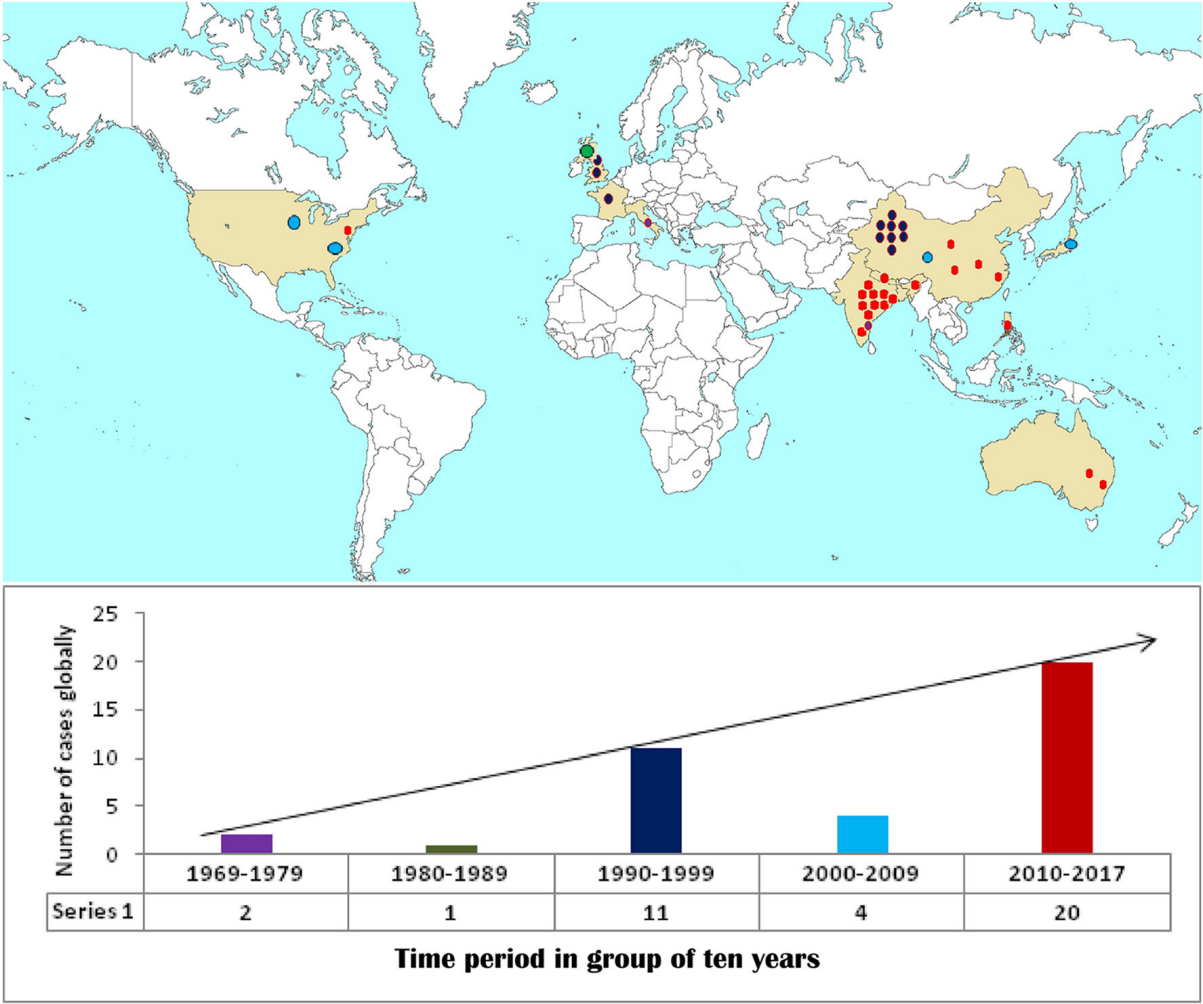
Time trend and geographical distribution of 38 cases of primary laryngeal aspergillosis in immunocompetent patients. Legend: https://commons.wikimedia.org/wiki/Maps_of_the_world

Laryngeal aspergillosis in the immunocompetent individual, though infrequently reported, seems to be an emerging condition. Lack of definite guidelines for clinical diagnosis due to the rarity of the disease might have resulted in under-reporting in the past. As shown in Fig. 2, the incidence of primary laryngeal aspergillosis in immunocompetent patients has been steadily rising over the past ten years. It seems that there has been a higher rate of reporting of the cases, especially after the 1990s, with a steady rise in the number of cases in the past seven years (Fig. 2). As depicted in the map, (Fig. 2) most of the new cases reported (11/20,) between 2010 and 2017, were from the Indian subcontinent, followed by China (4/20). This emphasizes that possibility of primary laryngeal aspergillosis must be entertained in all cases, presenting with typical features of laryngeal inflammation along with hoarseness of voice.

### Challenges in the diagnosis of laryngeal aspergillosis and utility of molecular diagnostics methods

As per the literature review, most of the laryngeal aspergillosis cases were diagnosed by the characteristic morphological features of the fungus in the biopsied material. However, result on species identification was lacking in majority of reported cases. Detection of hyphae, simulating those of *Aspergillus* in a biopsy specimen can be suggestive of fungal invasion but, is not necessarily pathognomonic of aspergillosis. Therefore, it becomes mandatory that the organism be isolated in pure culture and accurately identified. Few authors identified *Aspergillus* up to species level based on morphology and a couple of reports provided evidences of identification based on molecular methods [11, 12]. Moreover it is well known that fungal identification with conventional culture technique has its own limitations. As per the studies [13] conducted earlier, as well as in the present case, we could not successfully culture the fungus from the laryngeal biopsy. In these situations, etiological identification directly from clinical specimen via extraction of DNA and sequencing is advantageous. In this study, PCR on DNA extracted from paraffin-embedded tissue confirmed the aetiology. The extended region of the gene encoding the large ribosomal subunit (28S) of fungi was used for PCR amplification and sequencing. This region was previously explored for designing of broad range PCR primers and showed generation of successful amplicons and sequences from yeasts and filamentous fungi [14]. Because of the paucity of sequences of this extended region of fungal 28S rDNA in the public databases, the non D1/D2 region was rarely utilized for sequence-based detection and identification of fungi directly from clinical specimens. A recent study [15] showed the utility of the non D1/D2 region as a favorable target for the genus, and to a limited extent, species-level identification of pathogenic fungi in various fresh and FFPE samples. In the present study, attempt to amplify the internal transcribed spacer 1 (ITS1) region from the DNA extracted from the sample was not successful. One possible explanation might be due to the relatively larger size of the ITS1 region (~ 250–350 bp) than this non D1/D2 region (198 + _25 bp). Although accurate species identification required sequencing of at least a partial ITS region such as ITS 1 or ITS2, the non D1/D2 multicopy gene could give a satisfying genus level identification. In our study, this region could identify the genus and species of the pathogen with clear discrimination from other species of *Aspergilli* (with less % similarity scores) as evidenced from the BLAST hits. Therefore, this non D1/D2 region must be considered for PCR-sequencing from direct clinical specimens in those cases where partial ITS genes fail to amplify.

### Treatment of cases

In majority of the reported cases, including the present one, itraconazole was used as an empiric treatment, though voriconzole is the treatment of choice against invasive apsergillosis [16]. Possibly cost of the antifungal agent is an important limiting factor during treatment of fungal infections in developing countries. The critical condition of the patient, arising out of the acute laryngeal pathology may be a compelling reason for the empiric treatment on an emergency basis, yielding invariably positive outcome following therapy. Recent reports of the global emergence of azole resistance in *A. fumigatus* [17] *may be* of concern in the management of such patients in future. Prompt species identification and detection of resistance are of paramount importance in the management of laryngeal mycosis.

### Therapeutic outcome and relapse

In all 38 cases reviewed (Table 1), there was complete resolution of symptoms without any relapse, irrespective of the therapeutic modality adopted. There was not much difference in the time period between administration of antifungal drugs and relief of symptoms, regardless of whether the drug administered was itraconazole or voriconazole. Thus, considering the toxicity of conventional amphotericin B, and the cost of liposomal amphotericin B; empiric therapy with either itraconazole or voriconazole may be strongly advocated as better therapeutic options.

## Conclusion

Since last few decades cases of primary laryngeal aspergillosis in immunocompetent individuals are on the rise, globally. Patients responded to azoles with good prognosis. This is the first case of invasive laryngeal mycosis reported in Nepal. The extraction of DNA from tissue and sequencing helps to identify the etiological agent, when culture fails to isolate the fungus.

## Abbreviations

AMP-B: Amphotericin B
ATCC: American Type Culture Collection
COPD: Chronic obstructive pulmonary disease
DM: Diabetes mellitus
DNA: Deoxyribonucleic acid
FFPET: Formalin fixed paraffin embedded tissue
GERD: Gastroesophageal reflux disease
HE: Histopathological examination
HIV: Human immunodeficiency virus
ITCZ: Itraconazole
ITS: Internal transcribed spacer
PCR: Polymerase chain reaction
SEM: Scanning electron microscopy
VCZ: Voriconazole
VDRL: Venereal disease research laboratory test

## References

[1] Athanassiadou F, Kourti M, Papageorgiou T, Danielidis J (2005). Invasive aspergillosis of the larynx in a child with acute lymphoblastic leukemia. Pediatr Infect Dis J.

[2] Vrabec DP (1993). Fungal infections of the larynx. Otolaryngol Clin N Am.

[3] Dutta M, Jotdar A, Kundu S, Ghosh B, Mukhopadhyay S (2017). Primary laryngeal aspergillosis in the immunocompetent state: a clinical update. Braz J Otorhinolaryngol.

[4] Rao PB (1969). Aspergillosis of larynx. J Laryngol Otol.

[5] Lau A, Chen S, Sorrell T, Carter D, Malik R, Martin P, Halliday C (2007). Development and clinical application of a panfungal PCR assay to detect and identify fungal DNA in tissue specimens. J Clin Microbiol.

[6] Gangopadhyay M, Majumdar K, Bandyopadhyay A, Ghosh A (2014). Invasive primary aspergillosis of the larynx presenting as hoarseness and a chronic nonhealing laryngeal ulcer in an immunocompetent host: a rare entity. Ear Nose Throat J.

[7] Gallo A, Manciocco V, Simonelli M, Minni A, De Vincentiis M (2000). Clinical findings of laryngeal aspergillosis. Otolaryngol Head Neck Surg.

[8] Barnes RA, Rogers TR, Drouhet E, Cole GT, de Repentigny L, Latgé JP, Dupont B (1988). Aspergillosis in immunocompromised patients part I: the problem of diagnosis. Fungal Antigens.

[9] Vennewald I, Henker M, Klemm E, Seebacher C. Fungal colonization of the paranasal sinuses. Mycoses. 1999;42(Suppl 2):33–6. 10865901

[10] Darley D, Lowinger D, Plit M. Laryngeal aspergilloma: a complication of inhaled fluticasone therapy for asthma. Respirology Case Reports. 2014;2(4):123–5. 10.1002/rcr2.70. 10.1002/rcr2.70 PMC4263490 25530858

[11] Ran Y, Yang B, Liu S, Dai Y, Pang Z, Fan J, Bai H, Liu S (2008). Primary vocal cord aspergillosis caused by aspergillus fumigatus and molecular identification of the isolate. Med Mycol.

[12] Ran Y, Li L, Cao L, Dai Y, Wei B, Zhao Y, Liu Y, Bai H, Zhang C (2011). Primary vocal cord aspergillosis and scanning electron microscopical observation of the focus of infection. Mycoses.

[13] Liu YC, Zhou SH, Ling L (2010). Aetiological factors contributing to the development of primary laryngeal aspergillosis in immunocompetent patients. J Med Microbiol.

[14] Khot PD, Ko DL, Fredricks DN (2009). Sequencing and analysis of fungal rRNA operons for development of broad-range fungal PCR assays. Appl Environ Microbiol.

[15] Gade L, Hurst S, Balajee SA, Lockhart SR, Litvintseva AP. Detection of mucormycetes and other pathogenic fungi in formalin fixed paraffin embedded and fresh tissues using the extended region of 28S rDNA. Med Mycol. 2017;55(4):385–95. 10.1093/mmy/myw083. 10.1093/mmy/myw083 27630252

[16] Patterson TF, Thompson GR, Denning DW (2016). Practice guidelines for the diagnosis and Management of Aspergillosis: 2016 update by the Infectious Diseases Society of America. Clin Infect Dis.

[17] Jacques F. Meis, Anuradha Chowdhary, Johanna L. Rhodes, Matthew C. Fisher, Paul E. Verweij. Clinical implications of globally emerging azole resistance in Aspergillus fumigatus. Phil Trans R Soc B. 2016;371:20150460. 10.1098/rstb.2015.0460. Published 24 October 2016. 10.1098/rstb.2015.0460 PMC5095539 28080986

